# Positive Correlates of Sclerostin and Association with Peripheral Arterial Stiffness in Patients with Type 2 Diabetes Mellitus

**DOI:** 10.3390/medicina62040643

**Published:** 2026-03-27

**Authors:** Bang-Gee Hsu, Jer-Chuan Li, Du-An Wu, Ming-Chun Chen

**Affiliations:** 1 Division of Nephrology, Buddhist Tzu Chi General Hospital, Hualien 970473, Taiwan; gee.lily@msa.hinet.net; 2 School of Medicine, Tzu Chi University, Hualien 970374, Taiwan; despdu@yahoo.com.tw; 3 Division of Metabolism and Endocrinology, Buddhist Tzu Chi General Hospital, Hualien 970473, Taiwan; lijc.lijc@tzuchi.com.tw; 4 Department of Pediatrics, Hualien Tzu Chi Hospital, Buddhist Tzu Chi Medical Foundation, Hualien 970473, Taiwan

**Keywords:** sclerostin, dickkopf-1, brachial-ankle pulse wave velocity, peripheral arterial stiffness, diabetes mellitus

## Abstract

*Background and Objectives*: Sclerostin or dickkopf-1 (DKK1) inhibits the canonical Wnt/β-catenin signaling pathway, which regulates vascular calcification and may contribute to the development of arterial stiffness. The brachial–ankle pulse wave velocity (baPWV) measures peripheral arterial stiffness (PAS). This study aimed to investigate the correlation between sclerostin and DKK1 levels and PAS in patients with type 2 diabetes mellitus (T2DM). *Materials and Methods*: Biochemical data and sclerostin and DKK1 levels were analyzed in the fasting blood samples of 125 patients with T2DM. baPWV measurements using the VaSera VS-1000 automatic pulse wave analyzer classified patients with values > 18.0 m/s on either side into the PAS group. *Results*: Among patients with T2DM, 47 (37.6%) were classified as having PAS. These patients exhibited higher hypertension prevalence (*p* = 0.002); greater age (*p* < 0.001); elevated systolic (*p* < 0.001) and diastolic blood (*p* = 0.012) pressures; and increased fasting glucose (*p* = 0.001), glycated hemoglobin (*p* = 0.008), triglyceride (*p* = 0.001), blood urea nitrogen (*p* < 0.001), and creatinine (*p* = 0.001) levels, urine albumin-to-creatinine ratio (*p* = 0.039), and C-reactive protein (*p* = 0.024) and serum sclerostin (*p* < 0.001) levels, but decreased estimated glomerular filtration rate (*p* < 0.001). Multivariate logistic regression analysis identified serum sclerostin level (odds ratio, 1.127; 95% confidence interval, 1.058–1.200; *p* < 0.001) as an independent PAS predictor in patients with T2DM. Serum log-transformed sclerostin levels were positively correlated with left (*p* = 0.005) and right (*p* = 0.001) baPWV via Spearman’s rank-order correlation coefficient analysis. *Conclusions*: Serum sclerostin levels, but not DKK1 levels, are positively correlated with PAS in patients with T2DM.

## 1. Introduction

Type 2 diabetes mellitus (T2DM), a chronic metabolic disorder marked by insulin resistance-related hyperglycemia, poses a substantial global health challenge with increasing prevalence [1]. The International Diabetes Federation Diabetes Atlas estimated that 589 million adults were living with diabetes in 2024, and projections suggest that this number will continue to rise, reaching approximately 578 million by 2030 and more than 850 million by 2050 [2]. Among various T2DM complications, cardiovascular (CV) diseases remain the leading cause of death and disability in these patients [3]. Arterial stiffness (AS), a pathological condition of vascular damage, characterizes CV disease. Pulse wave velocity (PWV), a repeatable, noninvasive method for evaluating AS, reflects arterial elasticity clinically [4]. Brachial–ankle PWV (baPWV), which is simpler than carotid–femoral PWV, serves as a peripheral stiffness surrogate and CV risk marker in general, diabetic, and hypertensive populations [4,5]. Several meta-analyses, including recent studies in diabetic populations, have demonstrated that increased PWV is associated with higher risks of CV events, CV mortality, and all-cause mortality, highlighting arterial stiffness as an important predictor of adverse CV outcomes [6,7].

Identifying mechanisms behind T2DM-induced peripheral arterial stiffness (PAS) is critical. PAS pathogenesis in patients with T2DM encompasses multiple factors. Recent laboratory investigations confirm Wnt signaling’s contribution to vascular pathophysiology [8,9]. Sclerostin and dickkopf-1 (DKK1), both Wnt inhibitors, show increased expression during inflammatory and diabetic states [9]. Higher circulating sclerostin levels correlate with increased arterial stiffening in patients with stage 2–5 chronic kidney disease (CKD) and hypertension [10,11]. Moreover, elevated serum DKK1 levels have been correlated with endothelial dysfunction and vascular abnormalities, including in metabolic disorders such as in patients with T2DM [12]. In patients with T2DM, serum sclerostin levels are significantly elevated compared with healthy controls and independently associated with subclinical atherosclerosis assessed by carotid intima-media thickness (cIMT) [13]. Notably, 12 weeks of high-intensity interval training reduced serum sclerostin and DKK1 levels alongside cIMT improvements in patients with T2DM, further supporting the involvement of these Wnt inhibitors in diabetic vascular pathology [14]. Nevertheless, conflicting data exist regarding the association between serum sclerostin/DKK1 levels and PAS and arterial alterations [15]. Evidence regarding the associations between circulating sclerostin and DKK1 levels and AS remains limited, and the interaction between these Wnt signaling inhibitors in patients with T2DM has not been fully elucidated [16]. Thus, our study aimed to investigate the potential correlations between circulating sclerostin and DKK1 levels and PAS using baPWV measurements in patients with T2DM.

## 2. Materials and Methods

### 2.1. Participants

This study enrolled 125 individuals with T2DM who were diagnosed from January 2021 through July 2021 at an eastern Taiwan medical center. Blood pressure (BP) measurements followed standardized protocols, requiring participants to remain seated for ≥10 min before recording morning systolic BP (SBP) and diastolic BP (DBP) values. The exclusion criteria were cancer, active infections, cardiac failure, amputation, acute coronary events, thiazolidinedione/sodium glucose cotransporter 2 inhibitor use, and refusal of consent. Relevant clinical information, including medication use (antihypertensive agents, lipid-lowering drugs, and antidiabetic therapies) was recorded at enrollment. However, data on smoking status, diabetes duration, physical activity level, and detailed lifestyle variables were not systematically collected and thus could not be included in the analyses. Hualien Tzu Chi Hospital’s Research Ethics Committee granted approval (IRB108-219-A). All the participants provided written informed consent.

### 2.2. Anthropometric Analysis

Participants’ weight was measured to the nearest 0.5 kg in light clothing without shoes, whereas height was recorded to the nearest 0.5 cm. Waist measurements were taken at the narrowest point between the lowest ribs and hip bones, with the hands on the hips. Body mass index (BMI) was calculated as weight (kg) divided by height squared (m^2^) [1,17].

### 2.3. Biochemical Investigations

Fasting venous blood samples were collected and immediately centrifuged at 3000× *g* for 10 min. The separated serum was then aliquoted and stored at −80 °C until analysis. Serum biochemical parameters, including fasting glucose, glycated hemoglobin (HbA1c), blood urea nitrogen, creatinine, lipid profile components (total cholesterol, triglycerides, high-density lipoprotein cholesterol, and low-density lipoprotein cholesterol), total calcium, phosphorus, and C-reactive protein (CRP), were measured using an ADVIA 1800 autoanalyzer (Siemens Healthcare GmbH, Erlangen, Germany). Urinary albumin-to-creatinine ratio (UACR) was determined from random spot urine specimens [1,17]. The concentrations of serum sclerostin and DKK1 were measured using commercially available enzyme-linked immunosorbent assay kits obtained from Biomedica Immunoassays (Vienna, Austria) [14]. The intra-assay and inter-assay coefficients of variation were 4.5% and 6.2% for sclerostin, and 5.1% and 7.8% for DKK1, respectively. The assay sensitivity was 3.2 pmol/L for sclerostin and 1.7 pmol/L for DKK1, with detection ranges of 0–240 pmol/L and 0–160 pmol/L, respectively. We used the Chronic Kidney Disease Epidemiology Collaboration formula to compute the estimated glomerular filtration rate (eGFR).

### 2.4. Blood Pressure and Brachial–Ankle Pulse Wave Velocity Measurements

After venipuncture, patients assumed the supine position for 10 min prior to measurement. A trained operator obtained morning upper-arm BP readings via automated oscillometry, with three successive right brachial artery SBP and DBP measurements taken at 5 min intervals and averaged for analysis. Based on the Eighth Joint National Committee (JNC 8) standards [18], hypertension was defined as SBP ≥ 140 mmHg and DBP ≥ 90 mmHg or the use of antihypertensive medication within 2 weeks prior. BaPWV was measured using a volume plethysmographic apparatus (VP-2000, Omron, Kyoto, Japan) with four pneumatic cuffs containing plethysmographic and oscillometric sensors encircling both upper arms and ankles [19]. According to the Moens–Korteweg equation (PWV^2^ = E·h/r·*p*; where E = elastic modulus, h = vessel wall thickness, r = vessel radius, and *p* = blood density), PWV elevation reflects decreased vascular elasticity, increased wall thickness, or reduced luminal diameter [20]. The baPWV threshold of 18 m/s, established by Japan’s Physiological Diagnosis Criteria for Vascular Failure Committee based on recursive partitioning analyses, identifies hypertensive and high-risk CVD patients [20,21]. Patients with left or right baPWV exceeding this cutoff were designated as the PAS group.

### 2.5. Statistical Analyses

We performed a power analysis to determine whether our sample size was sufficient to detect statistically significant correlations between serum sclerostin levels and PAS in patients with T2DM. Because specific preliminary data investigating this association in T2DM populations are currently lacking, we based our expected effect size on previously published findings in hemodialysis patients [22]. Hemodialysis and T2DM patients share common pathophysiological mechanisms of accelerated vascular calcification and AS, making this a reasonable proxy. Furthermore, we powered our study to detect a medium correlation coefficient (*r* = 0.30), which is conventionally considered a clinically meaningful effect size for biomarker associations according to Cohen’s criteria. At an α of 0.05 and 80% statistical power, a minimum of 85 participants would be required [23]. Therefore, our enrolled sample size of 125 participants provided sufficient power (approximately 93%) to examine this relationship.

The normality of continuous variables was examined using the Kolmogorov–Smirnov test. Data with a normal distribution are expressed as mean ± standard deviation, and differences between groups were analyzed using the two-tailed independent Student’s *t*-test. Non-normally distributed parameters (TG, fasting glucose, HbA1c, BUN, creatinine, UACR, CRP, sclerostin, and DKK1) are shown as medians with interquartile ranges and were compared using the Mann–Whitney U test. Data expressed as the number of patients were analyzed by the χ^2^ test. The multivariate logistic regression model was constructed using a forced-entry method, in which variables significantly associated with PAS in univariate analyses (age, hypertension, SBP, DBP, and fasting glucose, HbA1c, TG, eGFR, UACR, and sclerostin levels) and considered clinically relevant were entered simultaneously. To assess potential multicollinearity, variance inflation factors were calculated for all covariates. Non-normally distributed data were logarithmically transformed to achieve normality for the subsequent analyses. Spearman’s rank-order correlation was used to evaluate the associations among serum log-transformed sclerostin (log-sclerostin), left/right baPWV, and other variables. The effect of sclerostin on PAS was assessed using receiver operating characteristic (ROC) curves and the area under the curve (AUC). Analyses were performed using SPSS (version 19.0; IBM Corp., Armonk, NY, USA), with *p* < 0.05 indicating statistical significance.

## 3. Results

Baseline characteristics, including demographic, clinical, laboratory, and medication data for the T2DM cohort, are summarized in Table 1.

Hypertension affected 59.2% (*n* = 74) of the patients, whereas PAS was observed in 37.6% (*n* = 47) of the patients. Compared with controls, the PAS group demonstrated advanced age (*p* < 0.001); impaired eGFR (*p* < 0.001); elevated SBP (*p* < 0.001), DBP (*p* = 0.012), and fasting glucose (*p* = 0.001), HbA1c (*p* = 0.008), TG (*p* = 0.001), BUN (*p* < 0.001), and creatinine (*p* = 0.001) levels; UACR (*p* = 0.039); and CRP (*p* = 0.024) and sclerostin (*p* < 0.001) levels; and higher hypertension prevalence (*p* = 0.002). No significant differences were observed in sex distribution, BMI, serum DKK1 level, or antihypertensive, antilipid, and antidiabetic medication usage patterns.

Consistent with the findings in Table 1, supplementary analyses based on baPWV quartiles showed significant trends across increasing baPWV categories, with higher age (*p* < 0.001), SBP (*p* < 0.001), triglyceride (*p* = 0.008), fasting glucose (*p* = 0.016), CRP (*p* = 0.017), hypertension prevalence (*p* = 0.004), BUN (*p* < 0.001), creatinine (*p* < 0.001), UACR (*p* = 0.019), and sclerostin (*p* < 0.001) levels, as well as lower eGFR (*p* < 0.001), in patients with higher baPWV quartiles (Supplementary Table S1).

After adjustment for covariates significantly associated with PAS, including age, hypertension, SBP, DBP, fasting glucose, HbA1c, TG, CRP, eGFR, UACR, and serum sclerostin levels, multivariate logistic regression analysis revealed several independent predictors of PAS in patients with T2DM. These included elevated serum sclerostin level (odds ratio [OR], 1.127; 95% confidence interval [CI], 1.058–1.200; *p* < 0.001), advanced age (OR, 1.099; 95% CI, 1.023–1.181; *p* = 0.010), increased SBP (OR, 1.096; 95% CI, 1.024–1.172; *p* = 0.008), higher fasting glucose level (OR, 1.018; 95% CI, 1.001–1.035; *p* = 0.034), and elevated CRP level (OR, 1.276; 95% CI, 1.077–1.513; *p* = 0.005, per 0.1 mg/dL increase) (Table 2). All variance inflation factors were below 4, indicating that multicollinearity was not a significant concern.

**Figure 1:**
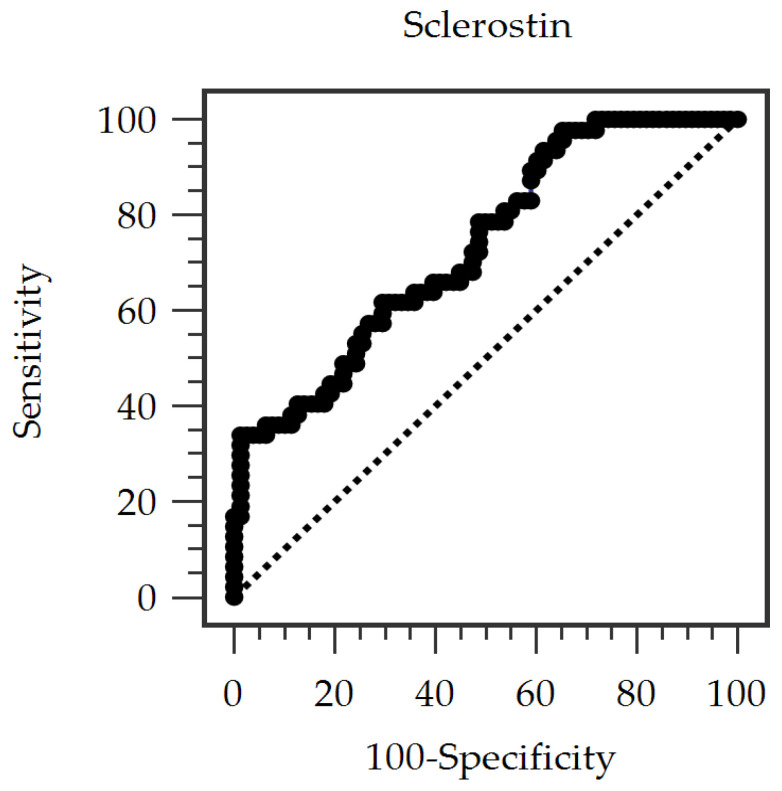
Receiver operating characteristic (ROC) curve of serum sclerostin for predicting peripheral arterial stiffness in patients with type 2 diabetes mellitus.

ROC curve analysis was performed to evaluate the predictive value of serum sclerostin levels for PAS. The optimal cutoff value was 43.97 pmol/L (Youden’s J statistic method), yielding 34.04% sensitivity, 98.72% specificity, 94.13% positive predictive value, and 71.30% negative predictive value. The AUC for predicting PAS based on serum sclerostin level was 0.733 (95% CI, 0.645–0.822; *p* < 0.001) (Figure 1).

Spearman’s rank correlation coefficient analysis (Table 3) was used to examine the associations among logarithmically transformed (log) sclerostin (log-sclerostin), left and right baPWV measurements, and clinical variables.

In patients with T2DM, serum log-sclerostin positively correlated with log-HbA1c (*r* = 0.207, *p* = 0.020) and log-creatinine (*r* = 0.336, *p* < 0.001) and negatively correlated with eGFR (*r* = −0.332, *p* < 0.001). Positive associations were observed between serum log-sclerostin levels and both left and right baPWV (*r* = 0.251, *p* = 0.005 and *r* = 0.287, *p* = 0.001, respectively). Both left and right baPWV demonstrated positive correlations with age (*r* = 0.547 and *r* = 0.573, both *p* < 0.001), SBP (*r* = 0.440 and *r* = 0.468, both *p* < 0.001), DBP (*r* = 0.237, *p* = 0.008 and *r* = 0.231, *p* = 0.009), and serum log-sclerostin (*r* = 0.251, *p* = 0.005 and *r* = 0.287, *p* < 0.001), log-TG (*r* = 0.229, *p* = 0.010 and *r* = 0.218, *p* = 0.015), log-glucose (*r* = 0.335, *p* < 0.001 and *r* = 0.269, *p* = 0.002), log-BUN (*r* = 0.342 and *r* = 0.332, both *p* < 0.001), log-creatinine (*r* = 0.430 and r = 0.398, both *p* < 0.001), log-UACR (*r* = 0.272, *p* = 0.003 and *r* = 0.204, *p* = 0.027), and log-CRP (*r* = 0.229, *p* = 0.010 and *r* = 0.267, *p* = 0.003) levels. Conversely, eGFR was negatively correlated with both left- and right-sided baPWV (*r* = −0.445 and −0.449, respectively; both *p* < 0.001) in patients with T2DM.

## 4. Discussion

In the present study, elevated serum sclerostin, advanced age, higher SBP, and increased fasting glucose and CRP levels emerged as independent determinants of PAS among T2DM patients. Spearman’s correlation analysis further showed that bilateral baPWV was positively correlated with age, SBP, DBP. It was also positively associated with several biochemical markers, including serum log-sclerostin, log-glucose, log-TG, log-BUN, log-creatinine, log-UACR, and log-CRP, and negatively correlated with eGFR. Serum log-sclerostin also correlated positively with log-HbA1c, log-creatinine, and bilateral baPWV, and negatively with eGFR. These findings indicate that higher serum sclerostin levels are independently associated with PAS in patients with T2DM; however, because of the cross-sectional design, no causal or mechanistic inference can be made.

AS is a degenerative vascular process characterized by low-grade inflammation and remodeling of collagen and elastin fibers. It is recognized as an independent marker of CV disease beyond traditional risk factors. PWV is a well-established, noninvasive tool for assessing arterial stiffness [24]. In particular, baPWV, a surrogate marker of large- to medium-sized artery stiffness, has demonstrated consistent prognostic value for CV events [25,26]. Inflammation and oxidative stress contribute to AS through both acute functional changes and chronic structural remodeling [20,27]. These processes impair arterial wall integrity and promote the progression of CV disease. Extensive research has consistently demonstrated that PWV is a robust and independent prognostic indicator of CV morbidity and mortality [6].

Arterial stiffening is influenced by multiple risk factors, including advanced age, hypertension, hyperglycemia, and renal impairment [20,28]. A meta-analysis revealed that age and BP were primary determinants of AS [20]. Age-associated SBP elevations are consistently reflected in baPWV measurements, revealing progressive vascular stiffening in both sexes [29]. The correlation between chronological progression and arterial rigidity indicates a fundamental physiological mechanism underlying CV aging dynamics. An epidemiological study demonstrated that SBP and TG levels independently predicted baPWV in 835 young adults. Furthermore, metabolic syndrome and its components (fasting glucose and BP) are significantly associated with the progression of AS in community-dwelling Taiwanese adults aged ≥ 40 years [30]. Hyperglycemia-related inflammation has been associated with more pronounced PAS. Diabetes-related advanced glycation end products have been linked to alterations in PWV, suggesting complex vascular pathophysiological interactions [31]. An incremental glucose peak and insulin resistance correlate with AS [32]. A recent study demonstrated early AS in Chinese patients with diabetes, with elevated post-load glucose levels closely associated with arterial elasticity changes measured by baPWV [32]. A meta-analysis further substantiated the robust association between the incidence of diabetes and accelerated vascular stiffness [33]. These studies have demonstrated consistent PWV elevation across diverse demographic parameters, highlighting the profound effects of diabetic conditions on arterial dynamics. Age and blood pressure are recognized as major determinants of AS, and baPWV is particularly susceptible to the hemodynamic influence of blood pressure at the time of measurement [20]. Therefore, SBP and DBP were retained in the multivariable model in addition to hypertension status to distinguish contemporaneous physiological effects from the presence of chronic hypertension. In addition, we evaluated multicollinearity using variance inflation factors, and all values were below 4, indicating that collinearity was not a major statistical concern in the final model.

Dyslipidemia-associated inflammation may be related to accelerated AS across age groups [34]. Fluvastatin treatment in patients with coronary artery disease and dyslipidemia can significantly reduce baPWV over a 5-year period, and this baPWV reduction directly correlates with decreased CRP levels [34]. CRP, a key inflammation marker, is associated with CV risk. In Japanese cohorts, increased CRP levels are correlated with elevated baPWV, along with age and SBP [35]. Renal dysfunction served as a confounder and amplifier of AS, which was evident even in the early stages of CKD. BaPWV correlates with renal outcomes in several studies [20,36]. A study of 145 patients with CKD stages 3–5 (baseline GFR, 29.9 mL/min/1.73 m^2^) linked baPWV to deterioration in renal function, including accelerated GFR decline, dialysis, and mortality [34]. Vascular calcification in advanced CKD may amplify AS [20]. Other studies have also reported that AS is associated with age, BMI, BP, antihypertensive use, and renal function, with baPWV values increasing as risks accumulate [4]. Consistent with prior literature, our findings corroborate that older age, hypertension, poor renal function, and elevated CRP, glucose, and TG values are associated with PAS in patients with T2DM and high baPWV.

Vascular pathophysiology in AS manifests through progressive fibrosis and calcification [37]. The Wnt/β-catenin signaling pathway is critically involved in the regulation of mesenchymal stem cell differentiation, vascular calcification, and inflammatory processes in the endothelium [17,37]. Wnt inhibitors, particularly sclerostin and DKK1, offer promising insights into understanding and potentially mitigating vascular calcification mechanisms [37]. Unlike DKK1, a broad Wnt pathway inhibitor, sclerostin demonstrates remarkable specificity for the canonical Wnt/β-catenin signaling pathway [15]. Generated by the SOST (sclerostin-encoding) gene in osteocytes, sclerostin has been associated with various vascular pathological processes, including endothelial dysfunction, vascular calcification, arterial stiffening, and atherosclerotic development, though the precise paracrine mechanisms remain to be fully established [8]. Sclerostin emerges as both a potential regulator and biomarker in vascular pathophysiology, demonstrating significant upregulation in calcifying smooth muscle cells. Circulating concentrations provide insights into the severity of vascular calcification [37]. Mechanistically, inhibition of canonical Wnt/β-catenin signaling by sclerostin may promote osteogenic differentiation of vascular smooth muscle cells, a key cellular process underlying vascular calcification and arterial remodeling. This phenotypic transition contributes to extracellular matrix mineralization and progressive arterial stiffening. Research by Leto et al. revealed elevated sclerostin levels in atherosclerotic plaques, which positively correlated with carotid intima-media thickness in postmenopausal women with T2DM [38]. The Wnt signaling pathway’s complex involvement in atherosclerosis mechanisms is further elucidated through Wnt3a’s anti-inflammatory properties, which suppress glycogen synthase kinase 3β and modulate NFκB-dependent gene transcription in macrophages [8]. Paradoxically, increased sclerostin levels have been hypothesized to interfere with these protective responses, which may be associated with atherosclerotic progression. Recent investigations also showed that high sclerostin concentrations were associated with accelerated AS, particularly when modulated by the renin–angiotensin system, in the general population, suggesting a sophisticated pathophysiological mechanism involving Wnt/β-catenin signaling and the renin–angiotensin system in vessel wall remodeling [39]. In addition, the ROC curve analysis in the present study demonstrated a moderate ability of serum sclerostin to discriminate PAS, suggesting its potential utility as an adjunctive biomarker for PAS in patients with T2DM. Nevertheless, the relatively low sensitivity observed in our analysis suggests that it is not suitable as a standalone screening tool for PAS and should be considered a complementary marker for risk stratification in selected clinical settings.

Epidemiological investigations have revealed elevated serum sclerostin levels as a significant independent biomarker of carotid–femoral PWV in patients on hemodialysis, particularly when parathyroid hormone levels remain <300 pg/mL [40]. Subsequent studies in patients with stage 2–5 CKD confirmed a positive association between circulating sclerostin levels and AS through multislice spiral computed tomography and PWV measurements [10]. Although the complex interplay among serum DKK1, endothelial dysfunction, and platelet activation was noted in patients with T2DM, most investigations have suggested a negative correlation between circulating DKK1 concentrations and vascular calcification, a fundamental pathological transformation in AS [15]. Greek [22] and Italian [41] studies have demonstrated sclerostin’s distinctive predictive potential, revealing its independent role as a CV events marker in patients on hemodialysis and a predictor of AS measured by PWV in ambulatory populations, consistently outperforming DKK1 after rigorous confounder adjustment. Postmenopausal women with an elevated PWV (>9 m/s) exhibit significantly higher sclerostin levels. Moreover, sclerostin is positively correlated with the aortic artery calcification score, whereas DKK1 level shows an inverse association, suggesting an equilibrium between these two Wnt inhibitors governing arterial calcification and stiffness [9]. In our study, patients with T2DM with high PAS demonstrated significantly elevated serum sclerostin levels, but not DKK1 levels, with sclerostin emerging as an independent predictor after multivariate analysis. The ROC profile observed in this study indicates that serum sclerostin may have greater utility for identifying individuals at a relatively high likelihood of PAS when elevated, rather than for excluding PAS in a screening setting. The absence of an association between DKK1 and PAS in our cohort may reflect fundamental biological differences between these two Wnt signaling inhibitors. Sclerostin is primarily derived from osteocytes and selectively targets canonical Wnt/β-catenin signaling. In contrast, DKK1 is produced by multiple cell types and exerts broader regulatory effects within the Wnt signaling network. These differences in cellular origin and signaling specificity may help explain the divergent associations observed in our study. However, this interpretation remains speculative and cannot be confirmed due to the cross-sectional design of the study.

Elevated sclerostin concentrations are associated with insulin resistance and progression of prediabetes and diabetes. Serum sclerostin levels have been positively correlated with the duration of T2DM and HbA1c level [42]. As mentioned above, hyperglycemia may induce AS via various pathogenic pathways [43]. Hyperglycemia-associated vascular inflammation and increased oxidative stress have been linked to elevated sclerostin levels, which, in turn, may be associated with a higher risk of arteriosclerosis, though longitudinal evidence is needed to confirm this relationship [15]. Serum sclerostin levels rise with CKD progression, doubling or quadrupling in end-stage renal disease compared with normal renal function populations [15]. Impaired renal clearance and enhanced production contribute to the increase in sclerostin levels [44]. Following renal transplantation, circulating sclerostin levels quickly decrease as renal function improves [45]. This phenomenon may be related to the restoration of renal clearance after transplantation, allowing more effective elimination of circulating sclerostin, which is known to accumulate in patients with advanced kidney dysfunction. Consistent with previous reports, serum sclerostin levels in our cohort were positively correlated with HbA1c and creatinine levels and negatively correlated with eGFR.

Our study has some limitations. First, the modest sample size and single-center cross-sectional design of this study may limit the generalizability of the findings and preclude the determination of a causal or temporal relationship between serum sclerostin levels and PAS in patients with T2DM. Therefore, the observed associations should not be interpreted as evidence of a mechanistic or causal effect. Prospective longitudinal and mechanistic studies are needed to clarify whether sclerostin has a causal role in the development or progression of AS. Second, several unassessed factors influencing baPWV, such as smoking status, physical activity, duration of diabetes, obesity, arrhythmia, and vascular anomalies (e.g., stenosis or aneurysm), were not systematically assessed, which may introduce bias and limit the predictive power of our findings [20,24]. Although antihypertensive, antilipid, and antidiabetic medication use did not differ significantly between groups (Table 1), a residual pharmacological influence on arterial stiffness cannot be entirely excluded. Third, although multivariate adjustment was performed, the relatively modest number of PAS events compared with the number of candidate predictors may have limited the stability of the regression estimates. Large-scale prospective studies are required to explore causality. Fourth, baPWV values are highly dependent on SBP, necessitating careful assessment of PAS [46]. With 60% of participants having hypertension, AS measurements may be inflated, creating confounding bias. Finally, although the 18 m/s baPWV cutoff was adopted according to established Japanese physiological diagnostic criteria [20,21], the lack of Taiwanese population-specific reference values represents an important limitation and may affect the external validity and clinical applicability of this threshold in our cohort. We have added supplementary analyses using quartiles of baPWV, which demonstrated consistent trends across increasing baPWV categories, thereby providing additional support for the overall associations observed in the main analysis. In addition, although serum sclerostin and DKK1 concentrations were determined using commercially available ELISA kits, the absence of duplicate measurements may have affected analytical precision and should be acknowledged when interpreting the results. Further research should establish age-related baPWV parameters in the Taiwanese population by incorporating sex and BP variables.

## 5. Conclusions

This study identified serum sclerostin, but not DKK1, as being independently associated with baPWV-measured PAS in patients with T2DM, suggesting that circulating sclerostin may represent a potential biomarker of arterial stiffness in this population. The PAS-independent predictors in patients with T2DM include age, SBP, fasting glucose, and CRP levels. Log-sclerostin is positively correlated with log-HbA1c, log-creatinine, and bilateral baPWV and negatively correlated with eGFR. Due to the cross-sectional design of the study, the observed findings should be interpreted as reflecting associations rather than causal relationships. Future prospective studies are warranted to investigate the temporal and causal association between sclerostin levels and PAS in patients with T2DM.

## Figures and Tables

**Table 1:** Clinical characteristics of patients with type 2 diabetes mellitus stratified by the presence or absence of peripheral arterial stiffness.

Characteristics	All Participants (*n* = 125)	Control Group (*n* = 78)	High PAS Group (*n* = 47)	*p*-Value
**Demographics and Anthropometrics**				
Age (years)	62.41 ± 12.72	58.98 ± 13.00	65.15 ± 9.97	<0.001 *
Male, *n* (%)	71 (56.8)	45 (57.7)	26 (55.3)	0.795
BMI (kg/m^2^)	26.61 ± 4.05	26.38 ± 4.13	26.99 ± 3.92	0.420
**Hemodynamic and Vascular Parameters**				
Left brachial–ankle PWV (m/s)	16.60 ± 2.99	14.87 ± 1.68	19.47 ± 2.42	<0.001 *
Right brachial–ankle PWV (m/s)	16.34 ± 2.90	14.68 ± 1.66	19.12 ± 2.33	<0.001 *
SBP (mmHg)	141.44 ± 20.05	134.87 ± 16.47	152.34 ± 20.85	<0.001 *
DBP (mmHg)	82.37 ± 10.68	80.53 ± 9.66	85.43 ± 11.65	0.012 *
Hypertension, *n* (%)	74 (59.2)	38 (48.7)	36 (76.6)	0.002 *
**Renal Function and Electrolytes**				
BUN (mg/dL)	16.00 (12.00–19.50)	14.00 (12.00–18.00)	18.00 (15.00–22.00)	<0.001 *
Creatinine (mg/dL)	0.9 (0.70–1.00)	0.80 (0.70–0.90)	1.00 (0.80–1.20)	0.001 *
eGFR (mL/min)	86.57 ± 26.45	94.77 ± 24.71	72.95 ± 23.66	<0.001 *
Calcium (mg/dL)	9.11 ± 0.44	9.12 ± 0.46	9.10 ± 0.40	0.818
Phosphorus (mg/dL)	3.57 ± 0.54	3.57 ± 0.52	3.57 ± 0.58	0.979
UACR (mg/g)	18.46 (7.45–99.07)	14.34 (6.44–57.73)	24.82 (9.73–212.89)	0.039 *
**Metabolic and Lipid Profiles**				
Glucose (mg/dL)	135.00 (119.00–170.50)	129.50 (113.75–147.25)	151.00 (128.00–214.00)	0.001 *
Glycated hemoglobin (%)	7.40 (6.60–8.85)	7.05 (6.50–8.30)	8.10 (6.90–9.40)	0.008 *
TCH (mg/dL)	163.48 ± 30.44	160.45 ± 30.79	168.51 ± 29.48	0.152
Triglyceride (mg/dL)	122.00 (87.00–176.00)	103.50 (74.25–173.75)	140.00 (114.00–178.00)	0.001 *
HDL-C (mg/dL)	46.38 ± 12.22	40.99 ± 11.99	45.36 ± 12.65	0.473
LDL-C (mg/dL)	100.93 ± 25.63	99.12 ± 25.27	103.94 ± 26.19	0.310
**Inflammatory and Bone Biomarkers**				
Sclerostin (pmol/L)	31.92 (24.43–39.68)	28.50 (21.63–36.24)	36.44 (28.73–52.25)	<0.001 *
Dickkopf-1 (pmol/L)	12.85 (9.16–17.73)	12.87 (8.75–17.62)	12.85 (9.71–18.95)	0.493
C-reactive protein (mg/dL)	0.09 (0.05–0.25)	0.07 (0.05–0.22)	0.15 (0.06–0.35)	0.024 *
**Medications**				
ARB use, *n* (%)	63 (50.4)	36 (46.2)	27 (57.4)	0.221
β-blocker use, *n* (%)	16 (12.8)	9 (11.5)	7 (14.9)	0.587
CCB use, *n* (%)	49 (39.2)	28 (35.9)	21 (44.7)	0.330
Statin use, *n* (%)	62 (49.6)	40 (51.3)	22 (46.8)	0.628
Fibrate use, *n* (%)	7 (5.6)	4 (5.1)	3 (6.4)	0.768
Metformin use, *n* (%)	75 (60.0)	49 (62.8)	26 (55.3)	0.407
Sulfonylurea use, *n* (%)	68 (54.4)	43 (55.1)	25 (53.2)	0.833
DPP-4 inhibitor use, *n* (%)	72 (57.6)	48 (61.5)	24 (51.1)	0.251
Insulin use, *n* (%)	44 (35.2)	25 (32.1)	19 (40.4)	0.342

Normally distributed continuous variables are presented as mean ± standard deviation and were compared using Student’s *t*-test, whereas non-normally distributed variables are expressed as median (interquartile range) and were analyzed using the Mann–Whitney U test. Categorical variables are shown as numbers (%) and were compared using the chi-square test. **Abbreviations:** PAS, peripheral arterial stiffness; BMI, body mass index; PWV, pulse wave velocity; SBP, systolic blood pressure; DBP, diastolic blood pressure; BUN, blood urea nitrogen; TCH, total cholesterol; HDL-C, high-density lipoprotein cholesterol; LDL-C, low-density lipoprotein cholesterol; eGFR, estimated glomerular filtration rate; UACR, urine albumin-to-creatinine ratio; ARB, angiotensin receptor blocker; CCB, calcium channel blocker; DPP-4, dipeptidyl peptidase-4. * Statistical significance was defined as *p* < 0.05.

**Table 2:** Factors associated with peripheral arterial stiffness in patients with type 2 diabetes mellitus: Multivariate logistic regression analysis.

Variables	Odds Ratio	95% Confidence Interval	*p*-Value
Sclerostin (pmol/L)	1.127	1.058–1.200	<0.001 *
Age (years)	1.099	1.023–1.181	0.010 *
SBP (mmHg)	1.096	1.024–1.172	0.008 *
Fasting glucose (mg/dL)	1.018	1.001–1.035	0.034 *
CRP (mg/dL) (per 0.1 increment)	1.276	1.077–1.513	0.005 *
Hypertension (present)	0.436	0.090–2.103	0.301
DBP (mmHg)	0.936	0.853–1.026	0.158
Glycated hemoglobin (%)	0.683	0.405–1.152	0.153
Triglyceride (mg/dL)	1.005	0.997–1.014	0.212
eGFR (mL/min)	0.983	0.954–1.014	0.276
UACR (mg/g)	1.000	0.999–1.000	0.118

Multivariate logistic regression analysis was performed after adjustment for age, hypertension, SBP, DBP, fasting glucose, glycated hemoglobin, triglycerides, CRP, eGFR, UACR, and serum sclerostin level. **Abbreviations:** SBP, systolic blood pressure; DBP, diastolic blood pressure; eGFR, estimated glomerular filtration rate; UACR, urine albumin-to-creatinine ratio. * Statistical significance was defined as *p* < 0.05.

**Table 3:** Correlations of left baPWV, right baPWV, and log-transformed sclerostin levels with clinical variables in patients with type 2 diabetes mellitus.

Variables	Left baPWV (m/s)		Right baPWV (m/s)		Log10 (Sclerostin) (pmol/L)	
	Spearman’s Coefficient of Correlation	*p*-Value	Spearman’s Coefficient of Correlation	*p*-Value	Spearman’s Coefficient of Correlation	*p*-Value
Age (years)	0.547	<0.001 *	0.573	<0.001 *	0.156	0.082
BMI (kg/m^2^)	0.120	0.184	0.074	0.413	−0.098	0.279
Left baPWV (m/s)	—	—	0.810	<0.001 *	0.251	0.005 *
Right baPWV (m/s)	0.810	<0.001 *	—	—	0.287	0.001 *
Log10 (Sclerostin) (pmol/L)	0.251	0.005 *	0.287	0.001 *	—	—
SBP (mmHg)	0.440	<0.001 *	0.468	<0.001 *	0.153	0.089
DBP (mmHg)	0.237	0.008 *	0.231	0.009 *	0.095	0.291
TCH (mg/dL)	0.085	0.349	0.119	0.188	−0.078	0.386
Log10 (TG) (mg/dL)	0.229	0.010 *	0.218	0.015 *	0.003	0.978
HDL-C (mg/dL)	−0.062	0.491	−0.061	0.498	−0.107	0.235
LDL-C (mg/dL)	0.008	0.933	0.036	0.692	−0.107	0.234
Log10 (Glucose) (mg/dL)	0.335	<0.001 *	0.269	0.002 *	0.095	0.293
Log10 (HbA1c) (%)	0.213	0.017 *	0.133	0.139	0.207	0.020 *
Log10 (BUN) (mg/dL)	0.342	<0.001 *	0.332	<0.001 *	0.132	0.143
Log10 (Creatinine) (mg/dL)	0.430	<0.001 *	0.398	<0.001 *	0.336	<0.001 *
eGFR (mL/min)	−0.445	<0.001 *	−0.449	<0.001 *	−0.332	<0.001 *
Log10 (UACR) (mg/g)	0.272	0.003 *	0.204	0.027 *	0.062	0.509
Total calcium (mg/dL)	−0.100	0.269	−0.094	0.296	−0.061	0.496
Phosphorus (mg/dL)	−0.079	0.378	−0.054	0.547	−0.092	0.308
Log10 (DKK1) (pmol/L)	0.022	0.811	0.037	0.686	−0.010	0.912
Log10 (CRP) (mg/dL)	0.229	0.010 *	0.267	0.003 *	−0.012	0.898

Triglyceride, glucose, HbA1c, BUN, creatinine, eGFR, UACR, DKK1, and sclerostin levels showed skewed distribution and were log-transformed before analysis. Data were analyzed using Spearman’s correlation analysis. **Abbreviations**: baPWV, brachial–ankle pulse wave velocity; BMI, body mass index; SBP, systolic blood pressure; DBP, diastolic blood pressure; TCH, total cholesterol; TG, triglyceride; HDL-C, high-density lipoprotein cholesterol; LDL-C, low-density lipoprotein cholesterol; HbA1c, glycated hemoglobin; BUN, blood urea nitrogen; eGFR, estimated glomerular filtration rate; UACR, urine albumin-to-creatinine ratio; DKK1, dickkopf-1; CRP, C-reactive protein. * Statistical significance was set at *p* < 0.05.

## Data Availability

The data presented in this study are available upon request from the corresponding author.

## Supplementary Materials

The following supporting information can be downloaded at: https://www.mdpi.com/article/10.3390/medicina62040643/s1, Table S1: Clinical characteristics according to quartiles of brachial–ankle pulse wave velocity in patients with type 2 diabetes mellitus.

## Abbreviations

The following abbreviations are used in this manuscript:

AS	Arterial stiffness
AUC	Area under the curve
baPWV	Brachial-ankle pulse wave velocity
BMI	Body mass index
BP	Blood pressure
BUN	Blood urea nitrogen
CI	Confidence interval
cIMT	Carotid intima-media thickness
CKD	Chronic kidney disease
CRP	C-reactive protein
CV	Cardiovascular
DBP	Diastolic blood pressure
DKK1	Dickkopf-1
eGFR	Estimated glomerular filtration rate
HbA1c	Glycated hemoglobin
OR	Odds ratio
PAS	Peripheral arterial stiffness
PWV	Pulse wave velocity
ROC	Receiver operating characteristic
SBP	Systolic blood pressure
T2DM	Type 2 diabetes mellitus
TG	Triglyceride
UACR	Urine albumin-to-creatinine ratio
