# Supplementary material for: Positive Correlates of Sclerostin and Association with Peripheral Arterial Stiffness in Patients with Type 2 Diabetes Mellitus
Source: Medicina (Kaunas). 2026 Mar 27;62(4):643. doi: 10.3390/medicina62040643 (PMC13117930; doi:10.3390/medicina62040643)
Supplement: Supplementary file 1 [file medicina-62-00643-s001.zip › medicina-4197111-supplementary.pdf]

Supplementary Table S1. Clinical characteristics according to quartiles of brachial-ankle pulse wave velocity in patients with type 2 diabetes mellitus.

Characteristics	baPWV (Q1 < 14.4 m/s) (*n* = 31)	baPWV (Q2 14.4-16.0 m/s) (*n* = 31)	baPWV (Q3 16.0-18.5 m/s) (*n* = 32)	baPWV (Q4 > 18.5 m/s) (*n* = 31)	*p* for trend
**Demographics and Anthropometrics**					
Age (years)	50.77 ± 13.39	63.32 ± 10.23	64.88 ± 9.40	70.58 ± 8.77	<0.001*
Male, *n* (%)	11 (35.5%)	19 (61.3%)	24 (75.0%)	17 (54.8%)	0.069
Body mass index (kg/m^2)	26.62 ± 4.59	25.86 ± 3.81	26.47 ± 3.54	27.51 ± 4.21	0.456
**Hemodynamic and Vascular Parameters**					
Left baPWV (m/s)	13.41 ± 1.47	15.53 ± 1.33	17.49 ± 1.78	19.93 ± 2.40	<0.001*
Right baPWV (m/s)	12.93 ± 0.93	15.14 ± 0.42	17.12 ± 0.83	20.18 ± 1.90	<0.001*
SBP (mmHg)	130.68 ± 20.28	137.58 ± 14.40	144.88 ± 18.23	152.52 ± 20.61	<0.001*
DBP (mmHg)	79.52 ± 10.32	81.13 ± 8.29	82.78 ± 9.80	86.03 ± 13.14	0.095
Hypertension, *n* (%)	13 (41.94%)	17 (54.84%)	20 (62.50%)	24 (77.42%)	0.004*
**Renal Function and Electrolytes**					
Blood urea nitrogen (mg/dL)	13.00 (12.00-16.00)	15.00 (12.00-18.50)	16.00 (12.75-22.00)	18.00 (15.50-22.00)	<0.001*
Creatinine (mg/dL)	0.80 (0.60-0.90)	0.90 (0.80-0.95)	0.95 (0.70-1.10)	1.00 (0.80-1.25)	<0.001*
eGFR (mL/min)	105.15 ± 29.25	86.74 ± 17.22	83.19 ± 21.87	71.30 ± 25.20	<0.001*
Total calcium (mg/dL)	9.12 ± 0.54	9.08 ± 0.45	9.21 ± 0.35	9.04 ± 0.39	0.472
Phosphorus (mg/dL)	3.69 ± 0.56	3.56 ± 0.50	3.51 ± 0.50	3.54 ± 0.61	0.583
UACR (mg/g)	10.91 (4.51-43.99)	15.86 (7.33-42.50)	25.62 (13.11-105.76)	26.40 (9.39-199.63)	0.019*
**Metabolic and Lipid Profiles**					
Fasting glucose (mg/dL)	127.00 (115.00-144.50)	138.00 (117.50-160.00)	135.50 (119.25-177.50)	151.00 (127.00-201.00)	0.016*

Glycated hemoglobin (%)	7.30 (6.70-8.10)	7.20 (6.35-8.45)	7.65 (6.57-8.68)	8.00 (6.85-9.10)	0.156
Total cholesterol (mg/dL)	157.94 ± 31.66	164.77 ± 32.34	162.97 ± 27.50	168.26 ± 30.71	0.608
Triglyceride (mg/dL)	120.00 (76.50-154.00)	101.00 (76.00-184.50)	120.00 (91.50-152.00)	148.00 (116.50-196.00)	0.008*
HDL-C(mg/dL)	48.03 ± 12.70	47.26 ± 10.82	44.72 ± 12.41	45.55 ± 13.12	0.696
LDL-C(mg/dL)	98.23 ± 25.45	100.55 ± 27.68	103.81 ± 21.87	101.03 ± 28.10	0.863
**Inflammatory and Bone Biomarkers**					
Sclerostin (pmol/L)	27.77 (23.17-33.35)	32.29 (23.14-39.31)	31.12 (24.01-40.54)	36.39 (27.81-47.13)	<0.001*
Dickkopf-1 (pmol/L)	12.71 (8.71-18.28)	14.23 (9.83-16.83)	12.71 (8.40-18.96)	12.25 (9.81-17.06)	0.933
C-reactive protein (mg/dL)	0.07 (0.05-0.21)	0.08 (0.05-0.18)	0.08 (0.05-0.28)	0.18 (0.08-0.33)	0.017*
**Medications**					
ARB use, *n* (%)	14 (45.16%)	15 (48.39%)	13 (40.62%)	21 (67.74%)	0.138
β-blocker use, *n* (%)	2 (6.45%)	6 (19.35%)	3 (9.38%)	5 (16.13%)	0.481
CCB use, *n* (%)	8 (25.81%)	14 (45.16%)	16 (50.00%)	11 (35.48%)	0.383
Statin use, *n* (%)	17 (54.84%)	15 (48.39%)	15 (46.88%)	15 (48.39%)	0.602
Fibrate use, *n* (%)	3 (9.68%)	2 (6.45%)	0 (0.00%)	2 (6.45%)	0.378
Metformin use, *n* (%)	19 (61.29%)	17 (54.84%)	25 (78.12%)	14 (45.16%)	0.534
Sulfonylurea use, *n* (%)	15 (48.39%)	19 (61.29%)	19 (59.38%)	15 (48.39%)	0.965
DDP-4 inhibitor use, *n* (%)	18 (58.06%)	18 (58.06%)	19 (59.38%)	17 (54.84%)	0.834
Insulin use, *n* (%)	12 (38.71%)	10 (32.26%)	7 (21.88%)	15 (48.39%)	0.635

Measured values between groups (quartiles of baPWV) were assessed using a Jonckheere-Terpstra test for parameters with non-normal distribution, and one-way analysis of variance for normally distributed data, and categorical variables were compared using the Cochran-Armitage test for trend.

Abbreviations: baPWV, brachial-ankle pulse wave velocity; SBP, systolic blood pressure; DBP, diastolic blood pressure; HDL-C, high-density lipoprotein cholesterol; LDL-C, low-density lipoprotein cholesterol; eGFR, estimated glomerular filtration rate; UACR, urine albumin-to-creatinine ratio;

ACE, angiotensin-converting enzyme; ARB, angiotensin receptor blocker; CCB, calcium channel blocker; DDP-4, dipeptidyl peptidase 4. *$p < 0.05$ was considered statistically significant.
